# The impact of low back pain on mental health among healthcare workers

**DOI:** 10.1192/j.eurpsy.2023.934

**Published:** 2023-07-19

**Authors:** I. Sellami, A. Feki, A. Abbes, M. L. Masmoudi, K. Jmal Hammami, M. Hajjaji

**Affiliations:** 1 Occupational medecine; 2 Rheumatology, Hedi Chaker hospital, University of Sfax, Sfax, Tunisia

## Abstract

**Introduction:**

Low back pain (LBP) is common among healthcare workers (HCWs) caused by providing patient care by bending forward for long durations. Even when symptoms are short-term and not medically serious, LBP can be associated with psychological distress.

**Objectives:**

This study aimed to assess the impact of low back pain on mental health among HCWs.

**Methods:**

The study was conducted in a group of HCWs from Hedi Chaker hospital in Sfax, Tunisia. Data were gathered between March-August 2022 using a self-administered questionnaire including socio-professional characteristics, the Nordic musculoskeletal questionnaire and Kessler Psychological Distress Scale (K6).

**Results:**

Our study included 74 HCWs. The mean age was 39,3 ± 10,5 years. The average job tenure was 15,5 ± 11,2 years. According to the Nordic musculoskeletal questionnaire, 29.7% of participants had low back pain during the last 12 months. Thirty participants (17.6%) had low back pain during the last 7 days. The mean score of K6 was 5,4±4,8 (range = 0–22). The proportion of respondents with high levels of psychological distress (K6 score of 13 or greater) was 9.5 %. The presence of low back pain during the last 12 months and the last 7 days was significantly associated with a high score of K6 (p = 0.008 and p = 0.01 respectively).

**Conclusions:**

Low back pain was associated with psychological distress. Occupational health and safety programs should focus on building ergonomically safe working conditions to enhance the mental health of the HCWs.

**Disclosure of Interest:**

None Declared

